# Aloperine Inhibits ASFV via Regulating PRLR/JAK2 Signaling Pathway In Vitro

**DOI:** 10.3390/ijms25169083

**Published:** 2024-08-21

**Authors:** Renhao Geng, Hongxia Shao, Kun Qian, Hongjun Chen, Aijian Qin

**Affiliations:** 1 College of Veterinary Medicine, Yangzhou University, Yangzhou 225009, China; 18705279737@163.com (R.G.); hxshao@yzu.edu.cn (H.S.); 2 Jiangsu Co-Innovation Center for Prevention and Control of Important Animal Infectious Diseases and Zoonoses, Yangzhou University, Yangzhou 225009, China; 3 Shanghai Veterinary Research Institute, Chinese Academy of Agricultural Sciences (CAAS), Shanghai 200241, China; vetchj@shvri.ac.cn

**Keywords:** African swine fever virus, aloperine, transcriptomics, PRLR, JAK2 signaling pathway

## Abstract

African swine fever (ASF) has become a global pandemic due to inadequate prevention and control measures, posing a significant threat to the swine industry. Despite the approval of a single vaccine in Vietnam, no antiviral drugs against the ASF virus (ASFV) are currently available. Aloperine (ALO), a quinolizidine alkaloid extracted from the seeds and leaves of bitter beans, exhibits various biological functions, including anti-inflammatory, anti-cancer, and antiviral activities. In this study, we found that ALO could inhibit ASFV replication in MA-104, PK-15, 3D4/21, and WSL cells in a dose-dependent manner without cytotoxicity at 100 μM. Furthermore, it was verified that ALO acted on the co- and post-infection stages of ASFV by time-of-addition assay, and inhibited viral internalization rather than directly inactivating the virus. Notably, RT-qPCR analysis indicated that ALO did not exert anti-inflammatory activity during ASFV infection. Additionally, gene ontology (GO) and KEGG pathway enrichment analyses of transcriptomic data revealed that ALO could inhibit ASFV replication via the PRLR/JAK2 signaling pathway. Together, these findings suggest that ALO effectively inhibits ASFV replication in vitro and provides a potential new target for developing anti-ASFV drugs.

## 1. Introduction

African swine fever (ASF) is an acute, severe, and hemorrhagic infectious disease that characterized by high morbidity and mortality rates in wild and domestic pigs [1]. Since its identification in Kenya in 1921 [2], ASF has spread to lots of countries worldwide, causing significant economic losses in the swine industry. In August 2018, ASF was introduced in China and rapidly spread across the country, resulting in substantial pig morbidity and mortality [3]. ASF is caused by ASFV, the sole member of the *Asfarviridae* family and only double-stranded DNA virus transmitted by arthropod vectors such as soft ticks [4,5]. The huge genome and complex structure of ASFV have presented significant challenges for vaccine development. Currently, only one approved vaccine for ASFV is available [6]. Therefore, whether we could develop safe and effective antiviral drugs to prevent the spread and outbreak of ASF is worthy of investigation.

Alkaloids are significant natural compounds widely distributed in nature, and most of them exhibit diverse biological activities due to their nitrogen-containing ring structures [7]. Some alkaloids have been found to exhibit anti-ASFV activity. For example, Tetrandrine and Berbamine hydrochloride (a bis benzylisoquinoline) were found to inhibit ASFV in a dose-dependent manner in vitro [8,9]. Aloperine (ALO) is a quinolizidine alkaloid extracted from the seeds and leaves of the medicinal plant *Sophora alopecuroides* L. [10]. ALO has been demonstrated to be a potent modulator of crucial signal pathways such as apoptosis, autophagy, cell cycle, PI3K/Akt/mTOR Signaling, NF-κB Signaling, Nrf2 Signaling, and Ras Signaling in multiple diseases [11]. In addition, ALO showed antiviral activity against various viruses, including Influenza A Virus (AIV), Human Immunodeficiency Virus (HIV), Ebola Virus (EBOV), Marburg Virus (MBV), Hepatitis B virus (HBV), and Hepatitis C virus (HCV) [7]. However, there are currently no reports on ALO against ASFV. In this study, we verified the anti-ASFV activity of ALO in vitro and explored the underlying mechanisms of its action.

## 2. Results

### 2.1. ALO Inhibited ASFV Replication In Vitro

**Figure 1:**
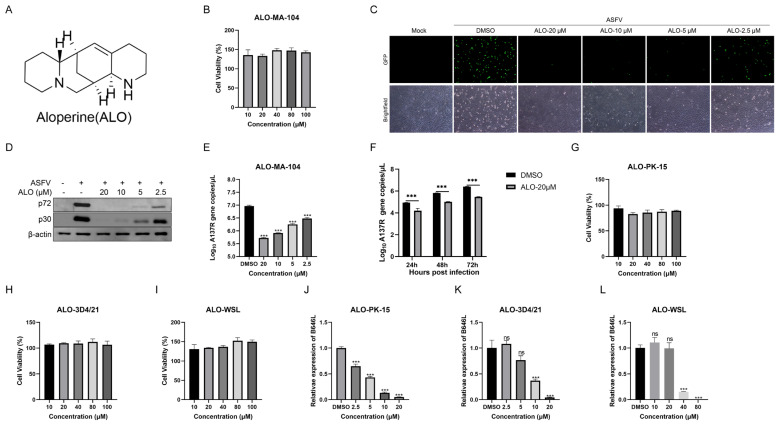
ALO inhibited ASFV replication in vitro. (**A**) Chemical structure of ALO. (**B**) The cytotoxicity of ALO in MA-104 cells was evaluated by CCK-8 assay after treatment for 48 h. (**C**–**E**) ASFV-infected MA-104 cells were treated with different concentrations of ALO (2.5, 5, 10, or 20 μM) for 24 h. The effect of ALO anti-ASFV was evaluated by fluorescence intensity, scale bar = 100 μm (**C**). The protein level of p72 and p30 was examined by Western blot (**D**), and the viral gene *A137R* copies (**E**) were determined by qPCR. (**F**) Viral gene *A137R* copies were detected by qPCR in ASFV-infected cells treated with ALO at different time points (24, 48, and 72 hpi). (**G**–**I**) The cytotoxicity of ALO in PK-15, 3D4/21, and WSL cells was evaluated by CCK-8 assay after treatment for 48 h. (**J**–**L**) The effect of ALO against ASFV in PK-15, 3D4/21, and WSL cells was determined by RT-qPCR. The data obtained from three independent experiments was analyzed using GraphPad Prism 8.0. Data. *** *p* < 0.001 and ns *p* > 0.05, compared to the DMSO control, respectively.

The cytotoxicity and anti-ASFV activity of ALO (Figure 1A) were evaluated in African green monkey cells MA-104. The CCK-8 assay showed that 100 μM was non-cytotoxic to the cells (Figure 1B). MA-104 cells treated with 2.5, 5, 10, or 20 μM ALO were infected with ASFVGZ for 24 h. The fluorescence images showed a significant decrease in fluorescence intensity in the ALO-treated group compared to the DMSO-treated group (Figure 1C). Western blot results revealed a dose-dependent reduction in the protein expression of p72 and p30 (Figure 1D). Similarly, as shown in Figure 1E, the qPCR results indicated that ALO significantly reduced viral gene copies of ASFV, with 20 μM ALO treatment reducing 1.24 log10 of *A137R* gene copies (*p* < 0.001). Moreover, as shown in Figure 1F, ALO could inhibit ASFV replication for at least 72 h post-infection (hpi). To verify the antiviral activity of ALO in porcine cells, similar experiments were performed on PK-15, 3D4/21, and WSL cells. The CCK-8 assay showed that 100 μM was also non-cytotoxic in these cells (Figure 1G–I). At 24 hpi, RT-qPCR results showed that ALO could inhibit the mRNA expression levels of the ASFV *B646L* gene in a dose-dependent manner (Figure 1J–L), although a higher concentration of ALO was required to exert ani-ASFV activity in WSL cells. These results suggested that ALO could effectively inhibit ASFV replication in vitro.

### 2.2. The Inhibition Stages of ALO on ASFV

**Figure 2:**
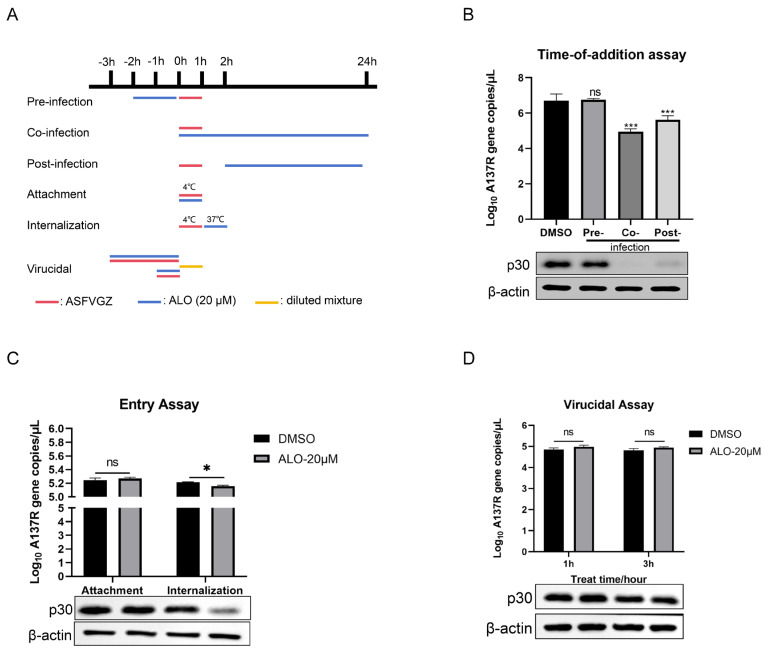
The inhibition stages of ALO on ASFV. (**A**) The schematic diagram for the process of ALO treatment at different stages of ASFV infection. (**B**) MA-104 cells were treated with ALO pre-, co-, or post-infection of ASFV. The samples were collected at 24 hpi and evaluated by qPCR and Western blot assay. (**C**) Effect of ALO treatment on the ASFV entry stage including attachment and internalization was determined by qPCR and Western blot assay. (**D**) Effect of ALO on ASFV inactivation after being treated for 1 h and 3 h, respectively, were determined by qPCR and Western blot assay. The samples were collected at 24 hpi and evaluated by qPCR and Western blot assay. * *p* < 0.05, *** *p* < 0.001, and ns *p* > 0.05, compared to the DMSO control, respectively.

To investigate which stage of ASFV replication ALO acted on, we designed and conducted time-of-addition experiments (Figure 2A). It was found that ALO could inhibit ASFV replication in co- and post-infection modes, leading to *A137R* gene copy reduction of 1.75 log10 and 1.08 log10 (*p* < 0.001), respectively, along with a decrease in p30 protein expression (Figure 2B). In addition, ALO was found to inhibit ASFV entry into cells by affecting the internalization stage rather than the attachment stage, causing *A137R* gene copy reduction of 0.06 log10 (*p* < 0.05) and p30 expression reduction (Figure 2C). Moreover, the virucidal assay results indicated that ALO had no virucidal effect on ASFV (Figure 2D).

### 2.3. ALO Did Not Exert Anti-Inflammatory Activity during ASFV Infection

**Figure 3:**
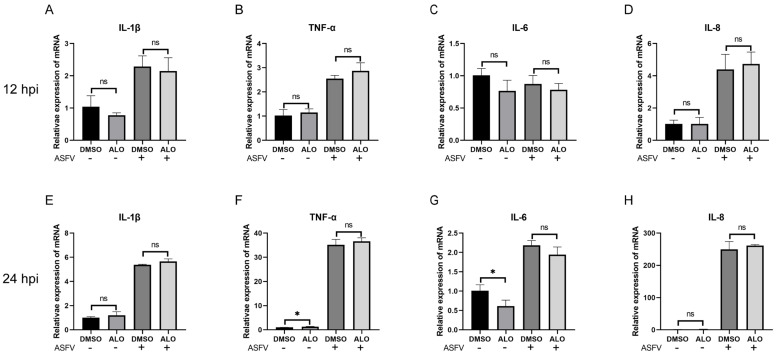
Effect of ALO on inflammatory cytokines. (**A**–**D**) RT-qPCR analysis of ASFV-infected MA-104 cells treated with 20 μM ALO; samples were collected at 12 hpi. (**E**–**H**) RT-qPCR analysis of ASFV-infected MA-104 cells treated with 20 μM ALO; samples were collected at 24 hpi. * *p* < 0.05 and ns *p* > 0.05 compared to the DMSO control, respectively.

Previous studies have demonstrated that ALO has anti-inflammatory properties [12,13,14]. Therefore, we investigated whether ALO exerted anti-inflammatory effects during ASFV infection. Here, we measured the expression levels of several inflammatory cytokines, such as IL-β, TNF-α, IL-6, and IL-8, induced by ASFV after ALO treatment at 12 and 24 hpi. RT-qPCR results showed that ASFV remarkably increased mRNA expression of these inflammatory cytokines, while ALO treatment did not result in a significant difference in cytokine expression compared to the DMSO treatment group (Figure 3A–H). These results indicated that ALO did not exert anti-inflammatory activity during ASFV infection.

### 2.4. Analysis of DEGs in MA-104 Cells Infected by ASFV Treated with/without ALO

**Figure 4:**
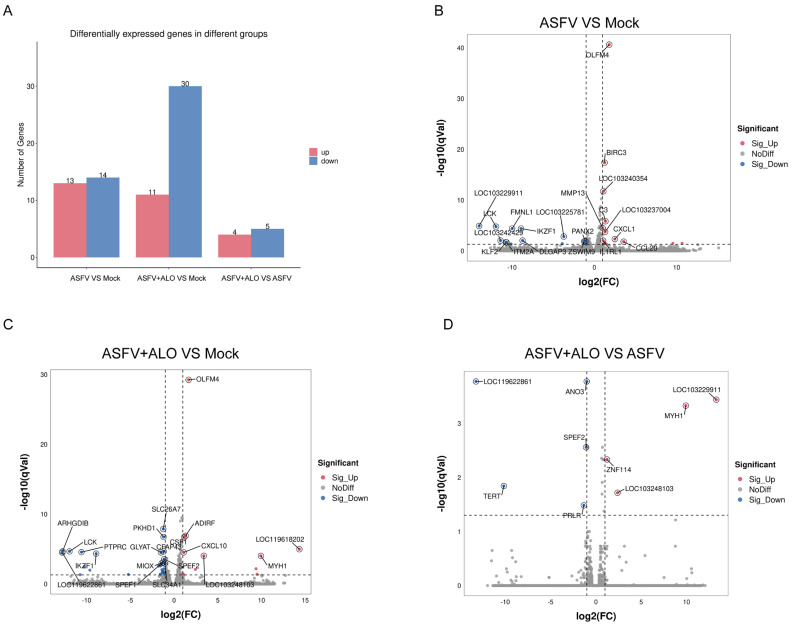
DEGs in MA-104 cells infected with ASFV treated with/without ALO. (**A**) The number of significantly up-regulated DEGs (log2FC ≥ 1 and q < 0.05) and down-regulated DEGs (log2FC ≤ −1 and q < 0.05) in each comparison group were counted. The most significant DEGs (top 20) in the groups of ASFV vs. Mock (**B**), ASFV + ALO vs. Mock (**C**), and ASFV + ALO vs. ASFV (**D**) were shown in the volcano maps.

To explore the anti-ASFV mechanisms of ALO, transcriptomic analysis of ASFV-infected cells treated with/without ALO was performed. The Pearson correlation coefficient among intra-group repeat samples was over 0.9, indicating that the sequencing data was reliable for further analysis. The results showed that there were 24,634 annotated genes identified in the three groups, with 13 significantly up-regulated and 14 significantly down-regulated DEGs between the ASFV-infected group and the Mock group. In parallel, the significantly up-regulated/down-regulated DEGs in the ALO-treated groups were 11/30 (ASFV + ALO vs. Mock) and 4/5 (ASFV + ALO vs. ASFV), respectively, as shown in Figure 4A. Moreover, the top 20 significantly up-regulated/down-regulated DEGs were shown in the volcano maps (Figure 4B–D).

**Figure 5:**
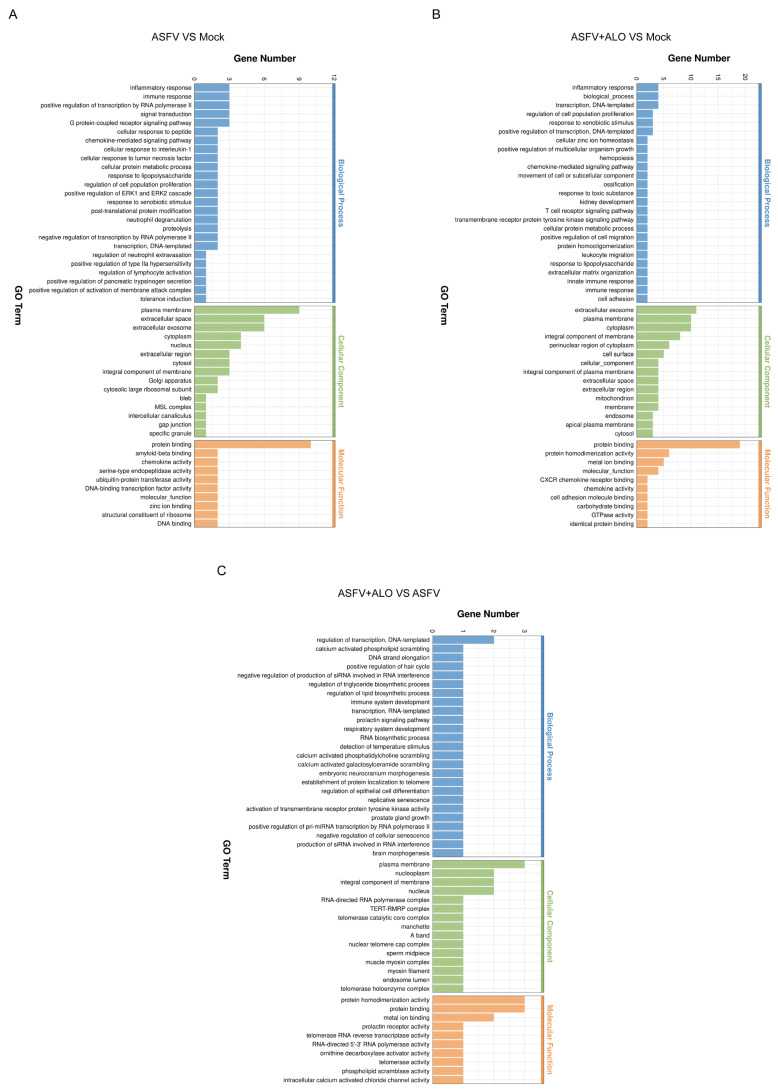
Gene Ontology (GO) terms enrichment of DEGs. The most significant enriched GO terms (top 50) among the DEGs in the groups of ASFV vs. Mock (**A**), ASFV + ALO vs. Mock (**B**), and ASFV + ALO vs. ASFV (**C**) were shown.

The GO and KEGG pathway enrichment analyses were conducted to classify and understand the functions of these DEGs. Compared with the Mock group, the top biological processes of DEGs in the ASFV group included inflammatory response, immune response, and signal transduction. The major cellular components of DEGs included plasma membrane, extracellular space, and extracellular exosome, while the primary molecular function was protein binding (Figure 5A). Notably, similar biological processes, major cellular components, and molecular functions were enriched in the ASFV + ALO group (Figure 5B). When compared with the ASFV group, the DEGs in the ASFV + ALO group were enriched in biological processes such as regulation of transcription, calcium-activated phospholipid scrambling, and the prolactin signaling pathway. The major cellular components included the plasma membrane, nucleoplasm, and integral components of the membrane. Additionally, the primary molecular functions included protein homodimerization activity and protein binding (Figure 5C).

**Figure 6:**
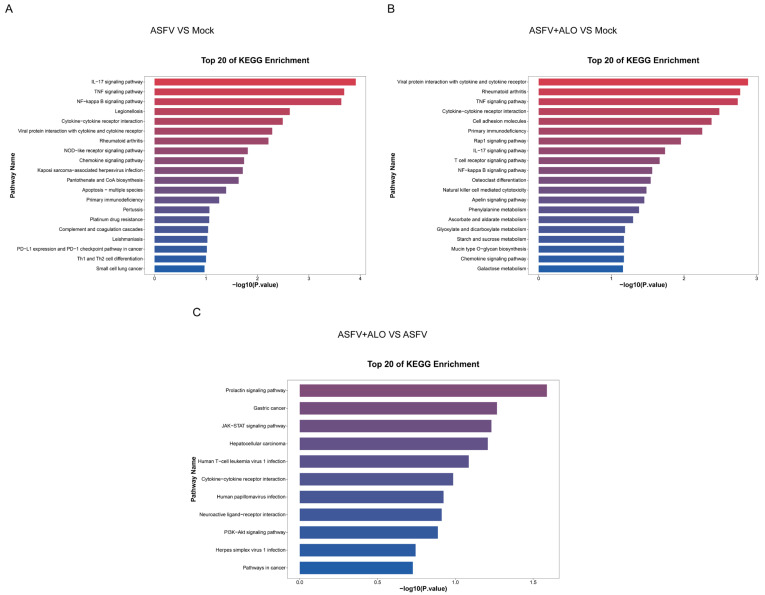
KEGG pathway enrichment of DEGs. The most significant enriched KEGG pathways (top 20) among the DEGs in the groups of ASFV vs. Mock (**A**), ASFV + ALO vs. Mock (**B**), and ASFV + ALO vs. ASFV (**C**) were shown.

For the KEGG analysis, a total of 97 pathways were enriched. Compared with the Mock group, inflammation-related signaling pathways, including the IL-17 signaling pathway, TNF signaling pathway, NF-kappa B signaling pathway, rheumatoid arthritis, and viral protein interaction with cytokine and cytokine receptor, were enriched in the ASFV group (Figure 6A). These pathways were also well-enriched in the ASFV + ALO group (Figure 6B). When compared with the ASFV group, the DEGs in the ASFV + ALO group were enriched in 11 signaling pathways, including the Prolactin signaling pathway, JAK-STAT signaling pathway, and PI3K-Akt signaling pathway (Figure 6C).

### 2.5. RT-qPCR Confirmation of the Selected DEGs

**Figure 7:**
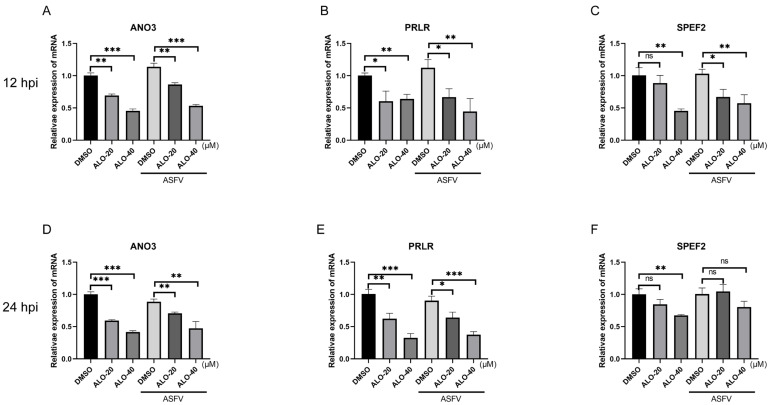
Validation of the selected DEGs. (**A**–**C**) RT-qPCR analysis of ASFV-infected MA-104 cells treated with 20 or 40 μM ALO, respectively; samples were collected at 12 hpi. (**D**–**F**) RT-qPCR analysis of ASFV-infected MA-104 cells treated with 20 or 40 μM ALO, respectively; samples were collected at 24 hpi. * *p* < 0.05, ** *p* < 0.01, *** *p* < 0.001, and ns *p* > 0.05, compared to the DMSO control, respectively.

In our analysis of DEGs across these three groups, we concentrated on the DEGs between the ASFV group and the ASFV + ALO group. Specifically, we selected three significantly downregulated DEGs—*ANO3* (Anoctamin-3), *PRLR* (Prolactin Receptor), and *SPEF2* (Sperm flagellar 2)—for validation using RT-qPCR analysis. As shown in Figure 7A–C, the mRNA expression levels of these genes were all downregulated in the ASFV + ALO group compared to the ASFV group at 12 hpi, consistent with the RNA-seq results. Additionally, when comparing the RT-qPCR results between the ALO group and Mock group, we observed that the mRNA expression levels of *ANO3* and *PRLR* were downregulated in the ALO group compared to the Mock group, whereas 20 μM ALO did not affect the mRNA expression of *SPEF2*. Furthermore, we investigated the inhibitory effects of ALO on these genes at 24 hpi. The mRNA expression levels of *ANO3* and *PRLR* were downregulated in the ASFV + ALO group compared to the ASFV group, while the mRNA expression of *SPEF2* showed no significant difference (Figure 7D–F). These results indicated that ALO could downregulate the expression of ANO3 and PRLR in a dose- and time-dependent manner.

### 2.6. Knockdown of PRLR Expression Could Inhibit ASFV Replication via Regulating JAK2 Signaling Pathway

**Figure 8:**
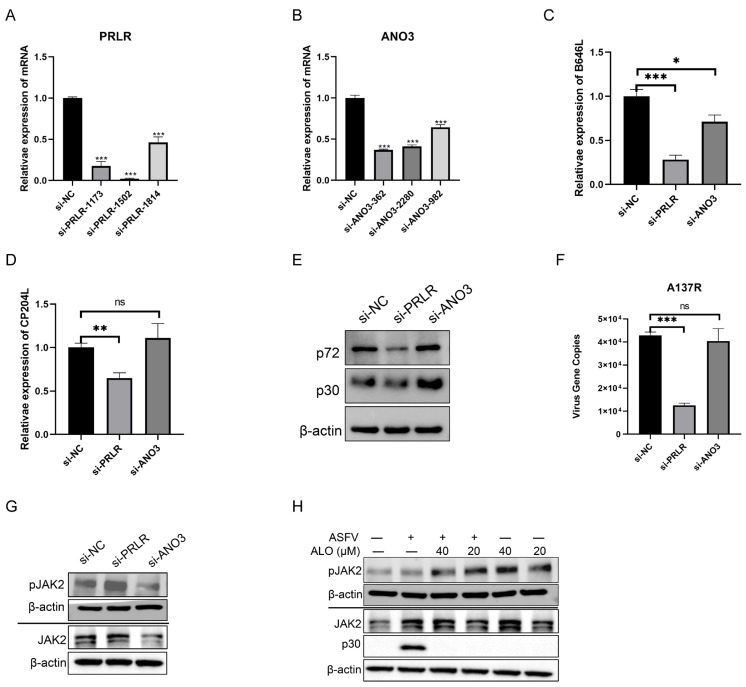
ASFV replication could be inhibited by the knockdown of PRLR expression via regulating the JAK2 signaling pathway. (**A**,**B**) MA-104 cells were transfected with siRNA negative control (si-NC), siRNAs targeting PRLR (si-PRLR), and ANO3 (si-ANO3) for 24 h, respectively. The mRNA levels of *PRLR* (**A**) and *ANO3* (**B**) were detected by RT-qPCR. (**C**–**G**) MA-104 cells were transfected with si-NC, si-PRLR-1502, and si-ANO3-362 for 24 h, respectively. Then, the cells were infected with ASFVGZ for 24 h. The mRNA levels of *B646L* (**C**) and *CP204L* (**D**) were detected by RT-qPCR. The protein levels of p72 and p30 were detected by Western blot (**E**). The viral gene *A137R* copies were determined by qPCR (**F**), and the protein level of pJAK2 (**G**) was detected by Western blot. (**H**) Western blotting of ASFV-infected MA-104 cells treated with either 20 or 40 μM ALO; samples were collected at 24 hpi. * *p* < 0.05, ** *p* < 0.01, *** *p* < 0.001, and ns *p* > 0.05, compared to the DMSO control, respectively.

Since ALO could downregulate the expression of ANO3 and PRLR, we investigated the effect of knocking down ANO3 and PRLR expression on ASFV replication. We screened siRNAs targeting PRLR and ANO3 using RT-qPCR, identifying si-PRLR-1502 and si-ANO3-362 as effective (Figure 8A,B). MA-104 cells were transfected with si-PRLR and si-ANO3 for 24 h and then infected with ASFVGZ (0.5 TCID50/cell). The effect of siRNA on ASFV replication was determined at 24 hpi. The results showed that the knockdown of PRLR expression, but not ANO3, could inhibit ASFV replication. This was shown by the downregulation of mRNA and protein expression of p72 and p30 and a reduction in *A137R* gene copies compared to the control group (Figure 8C–F). PRLR has been reported to regulate the JAK2 signal pathways [15,16]. Therefore, we detected whether the knockdown of PRLR expression and ALO treatment influenced the JAK2 signal pathway. Western blot results showed that the knockdown of PRLR expression and ALO treatment could increase the expression of phosphorylated JAK2. These findings indicated that ALO could inhibit ASFV replication via the PRLR/JAK2 signal pathway.

## 3. Discussion

Due to the lack of effective vaccines and antiviral strategies, ASF has caused severe losses to the global swine industry. Therefore, while developing vaccines, it is urgent to develop new anti-ASFV drugs. In addition, there are not many cell lines that can support the replication of ASFV. MA-104 cells have been reported to be susceptible to ASFV, which can be used for the isolation of ASFV from clinical samples and research on ASFV infection-related biological processes [17,18,19,20]. We compared ASFV replication in different cell lines and found that ASFV grows much better in MA-104 cells compared to swine cell lines such as PK15, WSL, and 3D4/21 cells (Supplementary Figure S1). Thus, MA-104 cells were chosen for our research. In this study, we demonstrated that ALO significantly inhibited ASFV replication in a dose-dependent manner in vitro. Additionally, time-of-addition assays revealed that ALO acted during the co- and post-infection stages. Moreover, the virucidal and virus entry assays indicated that ALO could not inactivate ASFV but influenced the viral internalization.

It has been reported that ALO could impede the cellular entry of various viruses, including HCV, HIV, EBOV, MARV, and Severe Acute Respiratory Syndrome Coronavirus 2 (SARS-CoV-2) by suppressing the fusion of the virus–host cell membrane and the activity of Cathepsin B rather than Cathepsin L [21,22,23,24]. A previous investigation revealed that ASFV infection triggered a 5-fold upregulation of tissue Cathepsin S and a 1.5–2-fold upregulation of Cathepsin L at 4 hpi [25]. Research on ASFV intracellular transport identified the co-localization of virus particles and Cathepsin L [26]. Therefore, whether ALO influenced viral internalization by suppressing the activity of Cathepsin B in ASFV infection needs further confirmation.

ALO has anti-inflammatory and antioxidant bioactivities. For example, ALO mitigated allergic airway inflammation by modulating the NF-κB, MAPK, and Nrf2/HO-1 signaling pathways [27]. Additionally, it protected mice from DSS-induced colitis by inhibiting inflammation through the PP2A-mediated PI3K/Akt/mTOR signaling pathway [28]. Therefore, we investigated whether ALO exerted anti-inflammatory effects during ASFV infection. Unexpectedly, ALO did not reduce the mRNA levels of inflammatory cytokines, including IL-1β, TNF-α, IL-6, and IL-8 induced by ASFV infection. Moreover, through GO and KEGG enrichment analysis of the transcriptome data, we found that ASFV infection activated inflammation-related signaling pathways in MA-104 cells, such as the IL-17 signaling pathway, TNF signaling pathway, NF-κB signaling pathway, NOD-like signaling pathway, and chemokine signaling pathway, which was consistent with the reported transcriptome sequencing results [29]. Corresponding to the RT-qPCR results, the ASFV + ALO group also enriched inflammation-related signaling pathways, such as the IL-17 signaling pathway, TNF signaling pathway, NF-κB signaling pathway, and rheumatoid arthritis (inflammation). These results indicated that ALO did not exert antiviral effects by modulating inflammatory responses. Subsequently, the validation of the transcriptome sequencing results revealed that ALO downregulated the *PRLR* and *ANO3* genes in a dose- and time-dependent manner.

PRLR (Prolactin Receptor) is a receptor for prolactin and type I cytokine, which is associated with various physiological and pathological processes, including breast cancer, mammary gland development, reproductive regulation, and immune regulation [30]. PRLRs have the potential action as viral receptors, as they are widely distributed in many tissues and subject to ligand-promoted receptor endocytosis [31,32]. ANO3 (Anoctamin 3), belonging to TMEM16 proteins, is a cell membrane protein functioning as a Ca^2+^-dependent phospholipid scramblase [33]. Recently, it has been reported that the inhibition of ANO6 (TMEM16F) phospholipid scramblase ameliorated SARS-CoV-2 infection [34]. In this study, we found that the knockdown of PRLR expression could inhibit ASFV replication, while the knockdown of ANO3 expression did not. Moreover, both the knockdown of PRLR expression and ALO treatment could increase the phosphorylation level of JAK2 in MA-104 cells, but the impact on the downstream signaling of JAK2 needs further research. The homology of the porcine PRLR gene (NCBI Gene ID: 414916) and African green monkey PRLR gene (NCBI Gene ID: 103215118) is 80.8%, while the protein homology is 72.7%, indicating a high degree of conservation (Supplementary Figure S2). Notably, RNA-seq analysis of the pig genome revealed that the Reads Per Kilobase per Million mapped reads (RPKM) of *PRLR* in the kidney (7.892 ± 2.148) is much higher than in the lung (0.19 ± 0.174) [35], which may explain why ALO requires a higher concentration to exert anti-ASFV function in WSL cells compared to PK-15 cells. Overall, these findings provide a foundational basis for further research into the potential use of ALO as an anti-ASFV drug, although the inhibitory effects of ALO against ASFV have yet to be evaluated in vivo.

## 4. Materials and Methods

### 4.1. Cells, Virus, and Regents

African green monkey kidney epithelial cells MA-104 and porcine kidney cells PK-15 that were stored in our laboratory were cultured in Dulbecco’s modified Eagle medium (DMEM) (Thermo Fisher Scientific, Waltham, MA, USA) supplemented with 10% fetal bovine serum (FBS) (Thermo Fisher Scientific) [36]. WSL-R4 (supported by Professor Jun Han, China Agricultural University) and 3D4/21 (the immortalized pulmonary alveolar macrophages, supported by Professor Jianzhong Zhu, Yangzhou University) were cultured in RPMI 1640 medium supplemented with 10% fetal bovine serum. All the cells were cultured at 37 °C in a 5% CO_2_ incubator. The ASFV strain GZ2018 (GenBank accession number: MT496893.1, the open reading frame of MGF100-1R was replaced by an eGFP expression cassette, hereafter called ASFVGZ) was prepared in the previous study [37] and stored at −80 °C before use. All operations involving ASFV in this study were carried out in a biosafety level-3 (BSL-3) laboratory at Yangzhou University (Yangzhou, China).

Aloperine (HY-13516) was purchased from MedChemExpress (Monmouth Junction, NJ, USA). The following commercial antibodies were used: Phospho-JAK2 (ET1607-34) and JAK2 (ET1607-35) were purchased from Huabio (Hangzhou, China); β-actin (ab6267) was purchased from Abcam (Cambridge, UK); and horseradish peroxidase (HRP)-conjugated anti-rabbit and anti-mouse secondary antibody were purchased from Jackson (West Grove, PA, USA). Anti-p30 and anti-p72 antibodies were prepared and stored in our laboratory [38].

### 4.2. Cell Viability

Cell Counting Kit-8 (Vazyme, Nanjing, China) was used to determine cell viability. MA-104, PK-15, 3D4/21, or WSL cells in 96-well plates treated with serial dilutions of ALO for 48 h were incubated with 10 μL of Cell Counting reagent per well for 1.5 h at 37 °C in the incubator. Absorbance values at 450 nm were detected by ELx808 Enzyme Labeling Instrument (Biotek, Winooski, VT, USA).

### 4.3. Fluorescence Imaging

The cells were fixed with 4% paraformaldehyde (PFA) at room temperature for 15 min. Images were captured using an Olympus IX50 inverted microscope (Olympus, Tokyo, Japan).

### 4.4. Evaluating the Inhibiting Effect of ALO on ASFV

To investigate the antiviral activity of ALO against ASFV, MA-104 cells were treated with different concentrations of ALO (2.5, 5, 10, and 20 μM) and 1 TCID_50_/cell ASFVGZ for 1 h at 37 °C. Following this incubation, the solution was removed, and the cells were washed three times. The cells were then cultured in a fresh medium containing 1% FBS and the corresponding concentration of ALO. At 24 hpi, samples were collected and analyzed using qPCR and Western blot assays. The same procedure was employed on PK-15, 3D4/21, and WSL cells to verify the antiviral activity of ALO in diverse porcine cell lines. At 24 hpi, the infected cells were collected for RT-qPCR analysis.

### 4.5. Time-of-Addition Assay

MA-104 cells were grown in a 24-well cell culture plate with a seeding density of 2 × 10^5^ cells/well. Amounts of 20 μM ALO were added before, during, and after ASFVGZ infection. For pre-infection, the cells were treated with 20 μM ALO for 2 h. Then, the cells were infected with 1 TCID_50_/cell ASFVGZ after washing with 1× PBS. The solution was replaced with a fresh culture medium after 1 h. For co-infection, 1 TCID_50_/cell ASFVGZ and 20 μM ALO were added to the cells at the same time. The solution was discarded after 1 h, and a culture medium containing 20 μM ALO was added after washing with 1× PBS. For post-infection, the cells were infected with 1 TCID_50_/cell ASFVGZ, and 20 μM ALO was added at 2 hpi. The samples were collected and analyzed with qPCR and Western blot assays at 24 hpi.

### 4.6. Virucidal Assay and Virus Entry Assay

For the virucidal assay, 20 μΜ ALO and ASFVGZ (1 TCID_50_/cell) were incubated at 37 °C for 1 h and 3 h, respectively. Subsequently, the treated mixture was diluted 20-fold and then incubated with MA-104 cells for 1 h. At 24 hpi, the samples were collected and analyzed with qPCR and Western blot assays.

The virus entry assay involves viral attachment and internalization. For the attachment assay, MA-104 cells were treated with 1 TCID_50_/cell ASFVGZ and 20 μΜ ALO at 4 °C for 1 h. Then, the cells were washed with 1× PBS three times, and a fresh culture medium containing 1% FBS was added. For the internalization assay, MA-104 cells were incubated with ASFVGZ (1 TCID_50_/cell) at 4 °C for 1 h, then the supernatant was replaced with 20 μM ALO, and the cells were incubated at 37 °C for 1 h. Subsequently, the cells were washed with 1× PBS three times, and a fresh culture medium containing 1% FBS was added. At 24 hpi, the samples were collected and analyzed with qPCR and Western blot assays.

### 4.7. Transcriptomic Sequencing and Data Analysis

Cell samples from three groups were prepared for transcriptomic sequencing, including groups of non-treated MA-104 cells (Mock group), ASFVGZ (1 TCID_50_/cell) infection (ASFV group), and ASFVGZ (1 TCID_50_/cell) infection treated with 20 μM ALO (ASFV + ALO group). In each group, triple-independent repeats were set. At 12 hpi, the cells were collected by a TRIzol reagent (ThermoFisher, USA) and stored at −80 °C.

The total RNA of MA-104 cells from three groups was extracted using the TRIzol reagent (Invitrogen, Carlsbad, CA, USA) following the manufacturer’s procedure. LC Bio-Technology Co., Ltd. (Hangzhou, China) performed the RNA quantification, RNA library construction, and sequencing. The expression levels of all transcripts and perform expression abundance for mRNAs were estimated by calculating the FPKM (fragment per kilobase of transcript per million mapped reads) value using StringTie and Ballgown [39].

Gene differential expression analysis was performed using DESeq2 and edgeR [40]. Genes with a false discovery rate (FDR) below 0.05 and absolute fold change ≥2 were considered differentially expressed. Differentially expressed genes (DEGs) were then subjected to enrichment analysis of Gene Ontology (GO) terms and Kyoto Encyclopedia of Genes and Genomes (KEGG) pathways. GO enrichment and KEGG enrichment analysis were performed as described by LC Bio-Technology Co., Ltd. (https://www.lc-bio.cn/).

### 4.8. Knockdown of PRLR and ANO3 Expression with siRNA

SiRNAs targeting monkey PRLR and ANO3 were synthesized by Sangong Biotech (Shanghai, China). The sequences are listed in Table 1. MA-104 cells in 12-well plates reaching 80% confluence were transfected with si-NC, si-PRLR, or si-ANO3 using the TransIT-X2 Dynamic Delivery System (MIR 6000, Mirus, Madison, WI, USA). After transfection for 24 h, the cells were collected to determine the expression of PRLR and ANO3 using RT-qPCR.

### 4.9. Western Blotting

The cells grown in a 12-well plate were lysed using RIPA lysis buffer containing 1× ProtLytic Protease and Phosphatase Inhibitor Cocktail (New Cell & Molecular Biotech, Suzhou, China). A BCA Kit (Vazyme, Nanjing, China) was used to quantify the extracted proteins. The samples were separated using 8% or 12.5% SDS-PAGE. After protein transfer, the nitrocellulose membrane was blocked with 5% BSA at room temperature for 1 h. The membrane was subsequently incubated with primary antibodies overnight at 4 °C and then exposed to HRP-conjugated anti-mouse or anti-rabbit secondary antibodies at room temperature for 1 h. Imaging was performed using the Tanon-5200 Multi-Infrared Imaging System (Tanon, Shanghai, China).

### 4.10. qPCR and RT-qPCR

The DNA from the collected supernatant of MA-104 cells was extracted using the FastPure Cell/Tissue DNA Isolation Mini Kit (Vazyme, Nanjing, China). Viral gene copies in the cell supernatant were detected using the absolute quantification method established in a previous study [41]. The total RNA of treated MA-104, PK-15, and WSL cells was extracted using the FastPure Cell/Tissue Total RNA Isolation Kit V2 (Vazyme, Nanjing, China). Then, the HiScript III RT SuperMix for qPCR (Vazyme, Nanjing, China) was used to reverse transcribe the extracted RNA. RT-qPCR was performed using SYBR qPCR Master Mix (Vazyme, Nanjing, China) and the LightCycler system (Roche Diagnostics, Mannheim, Germany). The relative quantification of the target gene was calculated using the 2^−ΔΔCt^ method, with β-actin as the housekeeping gene. Three independent replicates were performed. The primer sequences used in this study are listed in Table 2.

### 4.11. Statistical Analysis

Data are expressed as means ± standard deviation (SD). Differences were determined using a one-way analysis of variance or Student’s *t*-test with GraphPad Prism 8.0 software. *p* value < 0.05 was considered statistically significant (* *p* < 0.05; ** *p* < 0.01; *** *p* < 0.001), while “ns” indicates non-significance.

## 5. Conclusions

In summary, ALO could inhibit the replication of ASFV in a dose-dependent manner in vitro. ALO was found to act on co- and post-infection stages of ASFV and the viral internalization phase. ALO did not inhibit ASFV replication by exerting anti-inflammatory activity. Further transcriptome sequencing results indicated that ALO inhibited ASFV replication by regulating the PRLR/JAK2 signaling pathway. These findings provide a new candidate compound and a potential target for developing drugs against ASFV.

## Figures and Tables

**Table 1:** The sequence of siRNA used in this study.

siRNA	Sequence (5′–3′)
siNC	UUCUCCGAACGUGUCACGUTT
siPRLR-1173	GGGCAAGUCUGAAGAACUATT
siPRLR-1814	CCGCUAAACCCUUGGAUUATT
siPRLR-1502	GCAUAAGCAUAGAAGGCAATT
siANO3-362	GCAGAGAGGCUGAAUAUAATT
siANO3-2280	GCUUAAAGGGAUAUGUCAATT
siANO3-982	UCAAGUAAGCCAAGAAAUUTT

**Table 2:** The sequence of primers used in this study.

Target	Sequence (5′–3′)
ASFV-*A137R*-F	GGACATCGAGTGGTATTAAAAGG
ASFV-*A137R*-R	TGGCCTGAAAGTCAACATTGA
*β-actin* (monkey)-F	TCGATCATGAAGTGCGACGTG
*β-actin* (monkey)-R	GTGATCTCCTTCTGCATCCTGTC
*IL-1β* (monkey)-F	TAGACCTCTGCCCTCTGGAT
*IL-1β* (monkey)-R	CTCCATGGCTACAACAACCG
*TNF-α* (monkey)-F	CTGCACTTTGGAGTGATCGG
*TNF-α* (monkey)-R	GCTACAGGCTTGTCACTTGG
*IL-6* (monkey)-F	GGAACGAAAGAGAAGCTCTA
*L-6* (monkey)-R	CTTGTGGAGACGGAGTTCA
*IL-8* (monkey)-F	AGCTCTGTGTGAAGGTGCAG
*IL-8* (monkey)-R	CAGAGCTCTCTTCCATCAGAAA
*GAPDH* (pig)-F	CAAGGCTGTGGGCAAGGTCATC
*GAPDH* (pig)-R	CACGAGGAAGCAAGCAGAGTCAG
ASFV-*B646L*-F	CTGCTCATGGTATCAATCTTATCGA
ASFV-*B646L*-R	GATACCACAAGATCAGCCGT
ASFV-*CP204L*-F	GAGGAGACGGAATCCTCAGC
ASFV-*CP204L*-R	GCAAGCATATACAGCTTGGAGT
*ANO3* (monkey)-F	CAGGAAAGCCTATTGTTATGACTG
*ANO3* (monkey)-R	CACAACTTTTGCAGGCCAGTT
*PRLR* (monkey)-F	GCTGAGTGGGAGACCCATTT
*PRLR* (monkey)-R	CCATGATCTGGTTTGCAGCG
*SPEF2* (monkey)-F	AAGAAAGCCAGGCAAGTGATCC
*SPEF2* (monkey)-R	TTGAGCACGCGTAGTGAGG

## Data Availability

The data that support the findings of this study are available from the corresponding author (A.Q.) upon reasonable request.

## Supplementary Materials

The following supporting information can be downloaded at: https://www.mdpi.com/article/10.3390/ijms25169083/s1.

## References

[1] Karger A., Pérez-Núñez D., Urquiza J., Hinojar P., Alonso C., Freitas F.B., Revilla Y., Le Potier M.-F., Montoya M. (2019). An update on African swine fever virology. Viruses.

[2] Gaudreault N.N., Madden D.W., Wilson W.C., Trujillo J.D., Richt J.A. (2020). African swine fever virus: An emerging DNA arbovirus. Front. Vet. Sci..

[3] Zhang R., Huang Y., Bao C., Jung Y., Xu J., Qian Y. (2019). Epidemiology of African swine fever and analysis of risk factors of its spread in China: An overview. Chin. J. Virol..

[4] Alonso C., Borca M., Dixon L., Revilla Y., Rodriguez F., Escribano J.M., Ictv Report C. (2018). ICTV Virus Taxonomy Profile: Asfarviridae. J. Gen. Virol..

[5] Kleiboeker S., Scoles G., Burrage T., Sur J.-H. (1999). African swine fever virus replication in the midgut epithelium is required for infection of Ornithodoros ticks. J. Virol..

[6] Tran X.H., Le T.T.P., Nguyen Q.H., Do T.T., Nguyen V.D., Gay C.G., Borca M.V., Gladue D.P. (2022). African swine fever virus vaccine candidate ASFV-G-Δ*I177L* efficiently protects European and native pig breeds against circulating Vietnamese field strain. Transbound. Emerg. Dis..

[7] Cheng Y., Rauf A., Pan X. (2022). Research Progress on the Natural Product Aloperine and Its Derivatives. Mini Rev. Med. Chem..

[8] Qian B., Hu Y., Liu C., Zheng D., Han X., Gong M., Zou Y., Zeng D., Liao K., Miao Y. (2024). Tetrandrine (TET) inhibits African swine fever virus entry into cells by blocking the PI3K/Akt pathway. Virus Res..

[9] Zhu J., Huang L., Gao F., Jian W., Chen H., Liao M., Qi W. (2022). Berbamine Hydrochloride Inhibits African Swine Fever Virus Infection In Vitro. Molecules.

[10] Tolkachev O.N., Monakhova T.E., Sheichenko V.I., Kabanov V.S., Fesenko O.G., Proskurnina N.F. (1975). Alkaloids of a new type from *Sophora alopecuroides* L. Chem. Nat. Compd..

[11] Tahir M., Ali S., Zhang W., Lv B., Qiu W., Wang J. (2022). Aloperine: A Potent Modulator of Crucial Biological Mechanisms in Multiple Diseases. Biomedicines.

[12] Wang C., Choi Y.H., Xian Z., Zheng M., Piao H., Yan G. (2018). Aloperine suppresses allergic airway inflammation through NF-κB, MAPK, and Nrf2/HO-1 signaling pathways in mice. Int. Immunopharmacol..

[13] Ye Y., Wang Y., Yang Y., Tao L. (2020). Aloperine suppresses LPS-induced macrophage activation through inhibiting the TLR4/NF-κB pathway. Inflamm. Res..

[14] Yuan X.Y., Ma H.M., Li R.Z., Wang R.Y., Liu W., Guo J.Y. (2011). Topical application of aloperine improves 2,4-dinitrofluorobenzene-induced atopic dermatitis-like skin lesions in NC/Nga mice. Eur. J. Pharmacol..

[15] Kavarthapu R., Anbazhagan R., Dufau M.L. (2021). Crosstalk between PRLR and EGFR/HER2 Signaling Pathways in Breast Cancer. Cancers.

[16] Clevenger C.V., Gadd S.L., Zheng J. (2009). New mechanisms for PRLr action in breast cancer. Trends Endocrinol. Metab..

[17] Rai A., Pruitt S., Ramirez-Medina E., Vuono E.A., Silva E., Velazquez-Salinas L., Carrillo C., Borca M.V., Gladue D.P. (2020). Identification of a Continuously Stable and Commercially Available Cell Line for the Identification of Infectious African Swine Fever Virus in Clinical Samples. Viruses.

[18] Rai A., Pruitt S., Ramirez-Medina E., Vuono E.A., Silva E., Velazquez-Salinas L., Carrillo C., Borca M.V., Gladue D.P. (2021). Detection and Quantification of African Swine Fever Virus in MA-104 Cells. Bio-Protocol.

[19] Chen X., Zheng J., Liu C., Li T., Wang X., Li X., Bao M., Li J., Huang L., Zhang Z. (2023). CD1d facilitates African swine fever virus entry into the host cells via clathrin-mediated endocytosis. Emerg. Microbes Infect..

[20] Chen X., Zheng J., Li T., Liu C., Bao M., Wang X., Li X., Li J., Huang L., Zhang Z. (2023). Coreceptor AXL Facilitates African Swine Fever Virus Entry via Apoptotic Mimicry. J. Virol..

[21] Dang Z., Xie H., Zhu L., Zhang Q., Li Z., Huang L., Chen C.H. (2017). Structure Optimization of Aloperine Derivatives as HIV-1 Entry Inhibitors. ACS Med. Chem. Lett..

[22] Lv X.Q., Zou L.L., Tan J.L., Li H., Li J.R., Liu N.N., Dong B., Song D.Q., Peng Z.G. (2020). Aloperine inhibits hepatitis C virus entry into cells by disturbing internalisation from endocytosis to the membrane fusion process. Eur. J. Pharmacol..

[23] Zhang X., Liu Q., Zhang N., Li Q.Q., Liu Z.D., Li Y.H., Gao L.M., Wang Y.C., Deng H.B., Song D.Q. (2018). Discovery and evolution of aloperine derivatives as novel anti-filovirus agents through targeting entry stage. Eur. J. Med. Chem..

[24] Wang K., Wu J.J., Xin Z., Zeng Q.X., Zhang N., Huang W.J., Tang S., Wang Y.X., Kong W.J., Wang Y.C. (2021). Discovery and evolution of 12N-substituted aloperine derivatives as anti-SARS-CoV-2 agents through targeting late entry stage. Bioorganic Chem..

[25] Zhang F., Hopwood P., Abrams C.C., Downing A., Murray F., Talbot R., Archibald A., Lowden S., Dixon L.K. (2006). Macrophage transcriptional responses following in vitro infection with a highly virulent African swine fever virus isolate. J. Virol..

[26] Hernáez B., Guerra M., Salas M.L., Andrés G. (2016). African Swine Fever Virus Undergoes Outer Envelope Disruption, Capsid Disassembly and Inner Envelope Fusion before Core Release from Multivesicular Endosomes. PLoS Pathog..

[27] Chang Z., Zhang P., Zhang M., Jun F., Hu Z., Yang J., Wu Y., Zhou R. (2019). Aloperine suppresses human pulmonary vascular smooth muscle cell proliferation via inhibiting inflammatory response. Chin. J. Physiol..

[28] Fu X., Sun F., Wang F., Zhang J., Zheng B., Zhong J., Yue T., Zheng X., Xu J.F., Wang C.Y. (2017). Aloperine Protects Mice against DSS-Induced Colitis by PP2A-Mediated PI3K/Akt/mTOR Signaling Suppression. Mediat. Inflamm..

[29] Gao Q., Yang Y., Feng Y., Quan W., Luo Y., Wang H., Zheng J., Chen X., Huang Z., Chen X. (2022). Effects of the NF-κB Signaling Pathway Inhibitor BAY11-7082 in the Replication of ASFV. Viruses.

[30] Lopez Vicchi F., Becu-Villalobos D. (2017). Prolactin: The Bright and the Dark Side. Endocrinology.

[31] Wallis M. (2021). Do some viruses use growth hormone, prolactin and their receptors to facilitate entry into cells?: Episodic evolution of hormones and receptors suggests host-virus arms races; related placental lactogens may provide protective viral decoys. Bioessays.

[32] Swaminathan G., Varghese B., Thangavel C., Carbone C.J., Plotnikov A., Kumar K.G., Jablonski E.M., Clevenger C.V., Goffin V., Deng L. (2008). Prolactin stimulates ubiquitination, initial internalization, and degradation of its receptor via catalytic activation of Janus kinase 2. J. Endocrinol..

[33] Pedemonte N., Galietta L.J. (2014). Structure and function of TMEM16 proteins (anoctamins). Physiol. Rev..

[34] Sim J.R., Shin D.H., Park P.G., Park S.H., Bae J.Y., Lee Y., Kang D.Y., Kim Y.J., Aum S., Noh S.H. (2022). Amelioration of SARS-CoV-2 infection by ANO6 phospholipid scramblase inhibition. Cell Rep..

[35] Li M., Chen L., Tian S., Lin Y., Tang Q., Zhou X., Li D., Yeung C.K.L., Che T., Jin L. (2017). Comprehensive variation discovery and recovery of missing sequence in the pig genome using multiple de novo assemblies. Genome Res..

[36] Yin D., Shi B., Geng R., Liu Y., Gong L., Shao H., Qian K., Chen H., Qin A. (2024). Function investigation of p11.5 in ASFV infection. Virol. Sin..

[37] Liu Y., Li Y., Xie Z., Ao Q., Di D., Yu W., Lv L., Zhong Q., Song Y., Liao X. (2021). Development and in vivo evaluation of MGF100-1R deletion mutant in an African swine fever virus Chinese strain. Vet. Microbiol..

[38] Yin D., Geng R., Shao H., Ye J., Qian K., Chen H., Qin A. (2022). Identification of novel linear epitopes in P72 protein of African swine fever virus recognized by monoclonal antibodies. Front. Microbiol..

[39] Pertea M., Pertea G.M., Antonescu C.M., Chang T.C., Mendell J.T., Salzberg S.L. (2015). StringTie enables improved reconstruction of a transcriptome from RNA-seq reads. Nat. Biotechnol..

[40] Love M.I., Huber W., Anders S. (2014). Moderated estimation of fold change and dispersion for RNA-seq data with DESeq2. Genome Biol..

[41] Yin D., Geng R., Lv H., Bao C., Shao H., Ye J., Qian K., Qin A. (2021). Development of real-time PCR based on *A137R* gene for the detection of African swine fever virus. Front. Vet. Sci..

