# Supplementary material for: Aloperine Inhibits ASFV via Regulating PRLR/JAK2 Signaling Pathway In Vitro
Source: Int J Mol Sci. 2024 Aug 21;25(16):9083. doi: 10.3390/ijms25169083 (PMC11354989; doi:10.3390/ijms25169083)
Supplement: Supplementary file 1 [file ijms-25-09083-s001.zip › ijms-3116579-supplementary.pdf]

## Supplementary Figure Legend

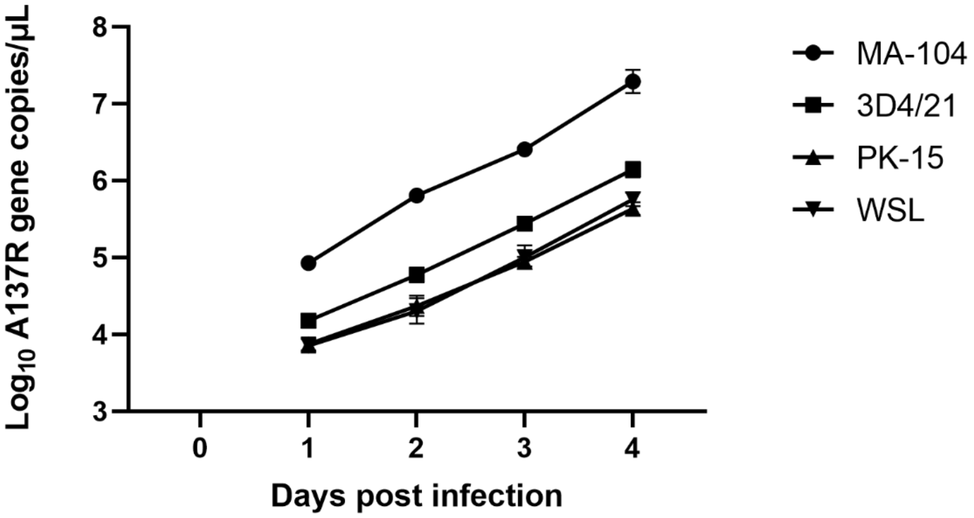


**Supplementary Figure S1.** Dynamic growth of ASFVGZ in different cell lines. MA-104, 3D4/21, PK-15 or WSL cells were infected with ASFVGZ (1 $TCID_{50}$/cell) and viral genome copies were calculated at the indicated times post-infection.

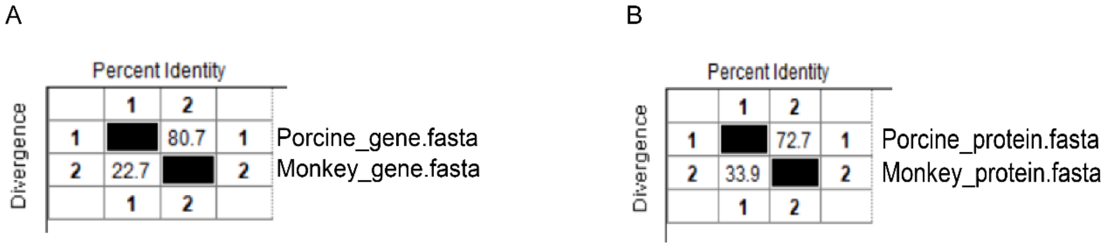


**Supplementary Figure S2.** Nucleotide (A) and protein (B) sequence homology of PRLR between pigs and African green monkeys was analyzed by the Clustal W method in MegAlign software.
